# Institutionalizing public health diplomacy: building trust in crisis

**DOI:** 10.3389/phrs.2026.1610349

**Published:** 2026-10-15

**Authors:** Olusimbo Ige, Dolapo Fakuade

**Affiliations:** 1 Institute of Medicine of Chicago, Chicago, IL, United States; 2 Chicago Department of Public Health, Chicago, IL, United States; 3 Coventry University, Coventry, United Kingdom

**Keywords:** crisis communication, diplomacy in public health, health communication strategies, public health leadership, public health systems

## Abstract

Public health crises are increasingly becoming diplomatic events. They require leaders to navigate scientific uncertainty while negotiating political, economic, social, and community interests. Yet public health preparedness continues to treat communication primarily as an individual leadership skill rather than as an institutional capacity for negotiation, consensus building, and trust. The COVID-19 pandemic exposed the consequences. Leaders were expected to communicate evolving evidence while absorbing public anger over difficult tradeoffs involving health, economic disruption, education, individual freedom, and equity. This commentary argues that crisis communication should be understood as public health diplomacy: a structured process through which leaders build trust, make values and tradeoffs transparent, engage diverse stakeholders, and adapt decisions as evidence changes. We propose three institutional practices: transparent decision-making frameworks, predefined reassessment checkpoints, and cross-sector advisory structures. We further argue that leadership turnover and chronic vacancies should be treated as indicators of system instability and emergency preparedness. Building public health diplomacy into training and crisis infrastructure can distribute responsibility for difficult decisions, strengthen institutional resilience, and better prepare leaders to protect health and trust when the next crisis arrives.

## Introduction

Public health crises manifest at the intersection of science, politics, economics, misinformation, and public trust. The COVID-19 pandemic made this tension impossible to ignore. Public health leaders were asked to contain a virus while minimizing economic disruption, educational loss, threats to individual freedom, and impact to health equity. Choosing one course of action often created harm elsewhere, yet leaders were expected to defend these choices as though they were purely scientific decisions.

This is more than a communication problem. It is a diplomacy problem. Public health leaders must negotiate among competing interests and values while communicating evidence that may change. The consequences of placing this burden on individuals are substantial. A survey of 583 US health departments found that 36% of public health officials left their positions during the first year of the pandemic; more than one-third of departures were associated with workplace harassment, bullying, or threats [1, 2]. The lesson is that public health institutions need better diplomatic infrastructure.

## Public health leaders are already diplomats

Diplomacy is often associated with foreign affairs, but its core functions of communication, negotiation, facilitation, consensus building, and managing competing interests are also central to public health practice. Local and national health leaders routinely negotiate with elected officials, health systems, businesses, educators, community organizations, journalists, scientists, and residents. During a crisis, these stakeholders may have very different definitions of acceptable risk.

Public health leadership experience in Chicago and New York, two of the largest cities in the US, Chicago and New York reinforced that it is often easier to get the science right than to navigate the consequences of acting on it. Decisions about schools, gatherings, scarce resources, and protective measures require epidemiologic expertise, but also the ability to explain uncertainty, negotiate disagreement, understand power, and maintain relationships with people who may oppose a decision.

Similar experiences in other countries reinforce this point. In South Africa, COVID-19 response structures emphasized coordinated, transparent, science-based, and agile leadership, supported by relationships across government, academia, the private sector, NGOs, and international partners [3]. In New Zealand, crisis leadership was communicated through a collective “team of five million” narrative that emphasized shared responsibility, care, trust, and cooperation [4, 5]. These experiences suggest that effective crisis leadership goes beyond what leaders know, it is also about how institutions create the conditions for collective action.

## From risk communication to public health diplomacy

Public health responses cannot wait for perfect information. But uncertainty should not become a reason for opaque decision-making. When evidence is evolving, a credible message may be: “Here is what we know, here is what we do not know, here is why we are acting, and here is what would cause us to change course.” This approach aligns with established principles of emergency risk communication and pandemic ethics: acknowledge uncertainty, explain the basis for decisions, and make tradeoffs visible [6–8].

## We recommend three intuitional practices to strengthen the diplomacy infrastructure

### Make decision transparency an institutional practice

Agencies should establish standing frameworks for weighing health risks alongside economic harm, educational disruption, individual autonomy, and equity [7, 8]. The framework should be public. Leaders may not be able to provide certainty, but they can explain the process: What evidence was considered? Which values were prioritized? What tradeoffs were recognized? Who participated?

Transparency does not eliminate disagreement, but it creates conditions for disagreement to be managed constructively. When people can see that decision-making recognizes their realities rather than reducing complex social choices to “the science,” trust becomes more possible.

### Build reassessment checkpoints into crisis decisions

Crisis measures should be treated as time-bound and revisable rather than open-ended. Before implementing a major intervention, agencies should identify the time horizon, the data to be monitored, and the conditions that would trigger modification or termination. For example: “We are implementing this measure for the next 14 days. We will review transmission and hospitalization data weekly and determine whether to extend, modify, or end it.”

Changing course in response to new evidence is not failure; it is responsible decision-making. Predefined reassessment points also turn disagreement from a contest over who was “right” into a shared discussion about what the evidence is revealing [6–8].

### Establish cross-sector structures for shared decision-making

Public health decisions should not be negotiated behind the walls of the health department. Business, education, government, community, and health leaders need mechanisms to discuss competing priorities before and during crises. South Africa’s experience illustrates the value of coordinated relationships across sectors, while also showing how top-down communication and weak links can impact trust [8]. Shared decision-making does not mean every stakeholder gets an equal vote on scientific questions. It means that the consequences of decisions are understood by the people who will experience and implement them. This is particularly important when misinformation is competing with credible information.

## The infodemic makes diplomacy more important

The modern public health leader operates in an information environment where credible evidence competes with misinformation, political narratives, commercial interests, and rapidly changing social media content. Trust cannot be built through information alone. It is built through relationships, consistency, listening, acknowledgment of uncertainty, and accountability.

The experience of New Zealand demonstrates how communication that is open, straightforward, motivational, and grounded in care can support trust and collective action [5]. At the continental level, African leadership during COVID-19 also demonstrated the importance of regional cooperation, locally informed leadership, and stronger African voice in global health decision-making [9]. Public health diplomacy therefore requires a broader competency set than traditional risk communication. It requires skills in facilitation, negotiation, building consensus, managing conflict, understanding power, and application of equity and ethical reasoning. These competencies belong in graduate education, executive development, and emergency preparedness exercises.

## Leadership stability is public health infrastructure

Without strengthening diplomatic skills and institutional practices, public health leaders are not able to thrive in their roles. Leadership turnover is not simply an administrative problem, it impacts system resilience. When experienced leaders leave, institutions lose relationships, institutional memory, community trust, and the capacity to navigate the next crisis. Evidence from Brazil demonstrates how political conflict contributed significantly to turnover among state health leaders [10]. In the US, since the pandemic, vocal, violent criticism of public health officials has continued to drive high-profile public health executive replacements at all levels of government [2].

If public health leaders are expected to act as diplomats, institutions must share the burden of diplomacy. This means protecting leaders from avoidable hostility by creating structures for transparent decision-making and building durable relationships across sectors and communities. Crisis preparedness requires stable leadership. Introducing new leadership in the midst of an emergency erodes community trust, as the foundational relationships required for effective response have not yet been established.

### Conclusion

Public health leadership during crisis requires navigating competing interests, managing uncertainty, building trust, and helping societies make difficult choices. That is diplomacy.

The field should move beyond training individual leaders to “communicate better” and build institutions that are better prepared to negotiate. Transparent decision frameworks, predefined reassessment checkpoints, cross-sector advisory structures, and deliberate investment in leadership stability can make difficult decisions more understandable, more inclusive, and more adaptable. If we expect public health leaders to serve as diplomats in the next crisis, we must give them the structures and institutional protection to do so.
